# Microbial, physiochemical and functional properties of probiotic yogurt containing *Lactobacillus acidophilus* and *Bifidobacterium bifidum* enriched by green tea aqueous extract

**DOI:** 10.1002/fsn3.2512

**Published:** 2021-08-05

**Authors:** Fatemeh Rahmani, Hassan Gandomi, Negin Noori, Azita Faraki, Melika Farzaneh

**Affiliations:** ^1^ Department of Food Hygiene Faculty of Veterinary Medicine University of Tehran Tehran Iran; ^2^ Department of Food Science and Technology Shahr‐e‐Qods Branch Islamic Azad University Tehran Iran

**Keywords:** antioxidant activity, green tea extract, probiotic bacteria, total phenol, yogurt

## Abstract

In this study, the effect of aqueous extract of green tea on the viability of probiotic bacteria including *Lactobacillus acidophilus* and *Bifidobacterium bifidum* and the sensory and physicochemical and functional properties of synbiotic yogurt was investigated during 4 weeks of storage. *L. acidophilus* and *B*. *bifidum* counts did not significantly change in yogurt containing 0.5% and 1% of the extract during storage. Also, the addition of the extract to yogurt highly increased the phenolic compounds, since the amount of phenolic compounds in yogurt containing 0.5% and 1% extract was 660 and 1,123 mg gallic acid/kg, respectively. In addition, a significant increase in the antioxidant activity of yogurt containing green tea extract was observed in comparison with the control. The amount of antioxidant activity increased during 4 weeks of storage, which reached to 4,193 and 7,337 mg BHT eq./kg in probiotic yogurt containing 0.5% and 1% extract, respectively. The acidity increased during 4 weeks of storage, while the pH decreased. Addition of the extract significantly increased the acidity of probiotic yogurt compared with the control (*p* < .05). In addition, in all studied groups, an increase in syneresis was observed during the study, and the syneresis was greater in yogurt containing aqueous extract of green tea, compared with the control group. Although adding the green tea extract to probiotic yogurt impaired taste, texture, and appearance compared with the plain yogurt, the overall acceptability of these samples was yet above the acceptable level. In conclusion, the results of the study showed that the addition of aqueous extract of green tea increased the antioxidant properties and the amount of phenolic compounds in yogurt, while the viability of probiotic bacteria was not changed. Therefore, the simultaneous use of green tea extract and probiotics in yogurt is recommended as an effective functional food formulation.

## Graphical abstract

1. Green tea extract fortification highly increased functionality of probiotic yogurt including antioxidant activity and total phenolic content. 2. Antioxidant activity of probiotic yogurt containing green tea extract increased during the storage. 3. No reduction in viability of probiotic *L. acidophilus* and *B. bifidum* was seen in synbiotic yogurt containing green tea extract during the study.

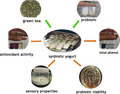

## 1 INTRODUCTION

Nowadays, functional foods have become one of the most important sectors of the modern food industry. It is well demonstrated that functional foods can offer potential health benefits by decreasing the risk of chronic disease beyond the widely accepted nutritional effects (Ishii et al., 2011). The most frequently used functional food are those contain probiotics, prebiotics, antioxidants, plant, calcium, and vitamins (Grajek et al., 2005). Probiotics are defined as live microorganisms which, when administered in adequate amounts, confer a health benefit to the consumers (FAO/WHO, 2001). There are a large number of probiotics used in dairy fermented foods, mainly in yogurts (Sanders, 1998; Ziemer & Gibson, 1998). The majority of probiotic bacteria belong to the *Lactobacillus* and *Bifidobacterium* genus, which are identified as safe (Holzapfel & Schillinger, 2002; Ouwehand et al., 1999). Prebiotics are nondigestible substances that provide a beneficial physiological effect on the host by selectively stimulating the favorable growth or activity of a limited number of indigenous bacteria (FAO/WHO, 2001). Modification of the human intestinal microbiota to balance probiotic species has become as main objective in nutritional science, and food producer has responded by expanding a range of functional food products, which contained prebiotics (Klaenhammer, 2000; Tannock et al., 2000). Synbiotic food preparations have both prebiotic compounds and probiotic bacteria (Shaghaghi et al., 2013). Yogurt and fermented dairy products have been welcomed by the community and the food industry as the best carriers for functional food ingredients including probiotics, prebiotics, and antioxidants such as polyphenols and carotenoids (O'Sullivan et al., 2016).

Tea (*Camellia sinensis L.)* is the second most popular drink, next to the water, consumed worldwide (Jaziri et al., 2009). It has three types including green (unfermented), white (partially fermented), and black (fermented) tea. There are many reports on the health benefits of tea consumption including reduction in cholesterol, prevention of neurodegenerative diseases, protection against cardiovascular disease, diabetes and liver diseases, antimutagenic properties, and protection against cancer (Najgebauer‐Lejko et al., 2014; Zuo et al., 2002). Green tea is a source of several phytochemicals that have beneficial physiological effects (Borrelli et al., 2004). The several great medicinal and health benefits of green tea that make it a valuable functional food is mainly associated with polyphenol compounds, which have high antioxidant properties (Bolling et al., 2009; Sharangi, 2009; Weisburger & Chung, 2002). Catechins are the primary polyphenol in tea that comprises 30%–42% of the dry weight in green tea leaves (Muniandy et al., 2016). Six major forms of catechins found in green tea include catechin, gallocatechin (GC), epigallocatechin (EGC), epigallocatechin‐3‐gallate (EGCG), epicatechin (EC), and epicatechin‐3‐gallate (ECG) (Graham, 1992). Green tea extracts are also incorporated into food preparations as potent antioxidants to enhance the shelf life, improve flavor, and give a healthier appeal to the consumer (Wang et al., 2003). Furthermore, it is reported that simultaneous consumption of green tea and dairy products including cheese and milk could protect integrity and antioxidant activity of the tea polyphenols during gastrointestinal digestion (Lamothe et al., 2014). As a response to consumer and food industry demand for functional yogurt, in this study, we aimed to evaluate the physicochemical (pH, acidity, total phenolic content, and syneresis), organoleptic (appearance, taste, texture, and overall acceptability), and antioxidant properties of yogurt incorporated with aqueous extract of green tea and probiotic *Lactobacillus acidophilus* and *Bifidobacterium bifidum* during 28 days refrigerated storage. The survival of probiotic bacteria during the storage was assayed as well.

## 2 MATERIAL AND METHODS

### 2.1 Preparation of aqueous extract of green tea

The green tea was prepared from farm in Gilan province, Iran. All the leaves were cut into small pieces and dried for two weeks in the shade at environmental temperature. It was then comminuted using a mechanical grinder (Moulinex). For extract preparation, 50 g of grinded plant was soaked in 450 ml water let shaking for 48 hr at 250 rpm, followed by filtration through filter paper of Whatman No. 1, then vaporized at 50°C using a rotary evaporator (Buchi Rotavapor R‐114, Switzerland), and further dried at 40°C. The extract powder was refrigerated at 4°C till running the experiments (Dahikar et al., 2010).

### 2.2 Preparation of probiotic bacteria

The probiotic bacteria including *L. acidophilus* (La5) and *B. bifidum* (Bb‐12) prepared from CHR Hansen (Horsholm, Denmark) was used in this study. Freeze‐dried bacteria were added to the sterile MRS broth medium and incubated for 48 hr at 37°C in aerobic condition and anaerobic jar for *L. acidophilus* and *B. bifidum*, respectively. Bacterial cultures were harvested by centrifugation at 4,000 × g at 4°C for 10 min and washed twice with sterile saline and collected by centrifugation. A bacterial suspension with optical density (OD) of 0.1 at 600 nm was prepared, and the cell numbers were determined using surface plate count technique through preparing serial dilutions and plating on MRS agar. The plates were then incubated at 37°C for 3 days in aerobic and anaerobic conditions for *L. acidophilus* and *B. bifidum*, respectively, as mentioned above. Bacterial number was calculated through counting bacterial colonies.

### 2.3 Production of yogurt

Standardized milk containing 1.5% fat and 12% dry matter was heat treated at 85°C for 15 min, then cooled down to 45°C. Direct vat yogurt starter culture containing *Lactobacillus delbrueckii sub‐species bulgaricus* and *Streptococcus thermophilus* (CHR Hansen) was added to the milk according to the manufacture instruction. For preparation of probiotic yogurt, 1% (v/v) probiotic bacterial suspension with 10^10^ cfu/ml density was inoculated. Aqueous extract of green tea (AEGT) at final concentrations of 0.5% and 1% (w/v) was added to the milk to prepare synbiotic yogurt. A plain yogurt without probiotic bacteria and AEGT was prepared as control. The yogurt samples were incubated at 42°C until the pH reached to 4.2–4.5. The fermentation was stopped by cooling the yogurt to 4°C. The prepared yogurt samples were stored at 4°C until analyzing at days 1, 7, 14, 21, and 28.

### 2.4 Measurement of pH and titratable acidity

The pH of the yogurt samples was measured using a digital pH meter (Jenway 3320, England). The titratable acidity (TA) was determined by titration method. Ten grams yogurt sample was mixed thoroughly with 90 ml of distilled water and titrated using 0.1 mol/L NaOH solution in the presence of phenolphthalein as indicator. Titratable acidity was expressed as % lactic acid.

### 2.5 Determination of total phenolic content

Total phenolic content of yogurt samples was measured by Folin–Ciocalteu method. Briefly, 5 g of yogurt samples was mixed with 15 ml distilled water in centrifuge tubes and centrifuged at 4,000 g for 10 min. A 0.1 ml aliquot of supernatant of each samples was mixed with 4 ml of 50% (v/v) Folin–Ciocalteau reagent and 2 ml of 2% sodium carbonate solution was added to the mixture and allowed to stand for 2 hr. The OD of the samples was read at 750 nm against solution consisting of 4 ml Folin–Ciocalteau and 2 ml sodium carbonate as blank. Gallic acid was used as standard to prepare calibration curve. The total phenolic content of the yogurt samples were expressed as mg gallic acid/kg yogurt (Shori & Baba, 2013).

### 2.6 Evaluation of antioxidant activity

The antioxidant activity of the yogurt samples was determined using DPPH (2,2‐diphenyl‐1‐picrylhydrazyl) free radical scavenging assay according to Brand–Williams et al. (1995) with minor modifications. Briefly, a 1/10 (w/v) dilution of yogurt sample in water was prepared. Then, an aliquot of 0.1 ml of each sample was added to 4 ml of DPPH solution with concentration of 0.004% in methanol. The mixture was shaken thoroughly and allowed to stand at room temperature for 30 min. The OD of the samples was read at 517 nm against water as blank. The inhibition percent (I%) of DPPH free radical was calculated. BHT was used as standard antioxidant, and the antioxidant activity of the yogurt samples was expressed as mg BHT equivalent/kg yogurt.

### 2.7 Measurement of the syneresis

Syneresis extent of the yogurt samples was measured using the centrifugal method according to Najgebauer–Lejko et al. (2014) and Sahan et al. (2008). Twenty grams of yogurt samples was prepared in centrifuge tubes and centrifuged at 4,000 × g for 15 min at 10°C. The syneresis extent was calculated as the weight percentage of whey released after centrifugation.

### 2.8 Evaluation of probiotic viability

The viable probiotic bacteria were quantified through surface plate count method. Ten grams of each yogurt samples was diluted with 90 ml of 0.1% peptone water. Preparation of serial dilutions, plating, and incubation conditions method was done as mentioned above. MRS bile agar and MRS agar containing 0.05% cysteine hydrochloride and 0.3% sodium propionate were used for selective enumeration of *L. acidophilus* and *B. bifidum*, respectively.

### 2.9 Sensory evaluation

Sensory analysis was performed using five‐point hedonic scale test (from dislike very much to like very much) by 10 trained assessors aged 20 and 40 years. Yogurt samples were randomly served at 7 to 10°C in plastic cups containing 50 ml of yogurt sample. Panelists completed a test assessment form for five sensory attributes including appearance, taste, texture, and overall acceptability.

### 2.10 Statistical analysis

All experiments were separately repeated three times. The data were expressed as means ± standard error of the mean (*SEM*). Statistical analysis was performed using one‐way analysis of variance (ANOVA) using SPSS 20 followed by Duncan's post hoc mean separation. Statistical significance was set at *p* < .05.

## 3 RESULTS

### 3.1 Viability of probiotic bacteria

The results of the count of *L. acidophilus* in probiotic yogurt containing the green tea extract (0.5% and 1%) are shown in Table 1. The probiotic count in treatment with no extracts was 7.8 log cfu/g in the first day, which decreased to 7.75 on day 28. At the end of the refrigerated storage period (day 28), *L. acidophilus* viable counts were 7.66 and 7.77 log cfu/g for probiotic yogurts containing 0.5% and 1% green tea extract, respectively. In general, there was no significant difference in count of *L. acidophilus* between different types of yogurt during 28 days (*p* > .05). The results of *B*. *bifidum* viability in probiotic yogurt containing different concentrations of green tea extract were demonstrated in Table 2. The viability of probiotic *B*. *bifidum* remained unchanged in all the treatment groups during the storage time. Furthermore, no statistically significant difference was seen in count of *B*. *bifidum* between different types of yogurt during 28 days (*p* > .05).

**TABLE 1:** Viability (log cfu/g) of *Lactobacillus acidophilus* in synbiotic yogurt containing aqueous extract of green tea during storage

Treatments	Storage time (day)				
	1	7	14	21	28
Probiotic	7.78 ± 0.08^a^	8.04 ± 0.15^a^	7.73 ± 0.38^a^	7.45 ± 0.18^a^	7.75 ± 0.22^a^
Probiotic + 0.5% extract	8.01 ± 0.21^a^	8.20 ± 0.05^a^	8.06 ± 0.37^a^	7.87 ± 0.17^a^	7.66 ± 0.26^a^
Probiotic + 1% extract	7.93 ± 0.01^a^	8.02 ± 0.01^a^	7.89 ± 0.09^a^	7.68 ± 0.35^a^	7.77 ± 0.12^a^

Note

Different superscript letters at each column show statistical significance between different treatments (*p* < .05).

**TABLE 2:** Viability (log cfu/g) of *Bifidobacterium bifidum* in synbiotic yogurt containing aqueous extract of green tea during storage

Treatments	Storage time (day)				
	1	7	14	21	28
Probiotic	7.81 ± 0.20^a^	8.09 ± 0.15^a^	7.80 ± 0.26^a^	7.50 ± 0.12^a^	7.82 ± 0.11^a^
Probiotic + 0.5% extract	8.05 ± 0.30^a^	8.15 ± 0.19^a^	8.0633 ± 0.15^a^	8.02 ± 0.11	8.01 ± 0.10^a^
Probiotic + 1% extract	7.90 ± 0.10	8.05 ± 0.06^a^	8.01 ± 0.13^a^	7.686 ± 0.42^a^	7.76 ± 0.08^a^

Note

Different superscript letters at each column show statistical significance between different treatments (*p* < .05).

### 3.2 Total phenolic content

**FIGURE 1:**
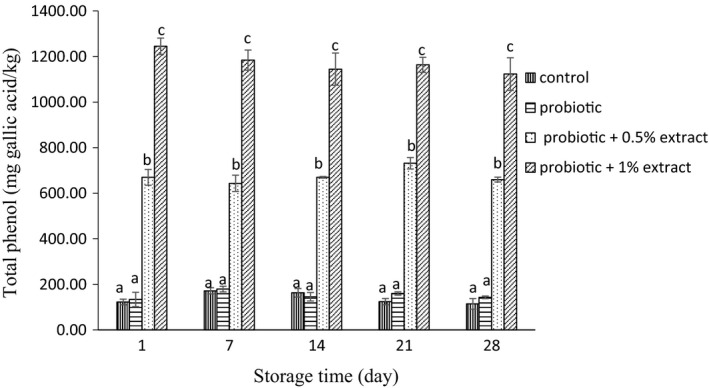
Total phenolic content of synbiotic yogurt containing aqueous extract of green tea during storage. Different letters at each day show statistical significance between different treatments (*p* < .05)

The phenolic content of the yogurt samples is indicated in Figure 1. The phenolic content of plain yogurt samples was 122.54 mg gallic acid/kg, which had no statistically significant difference (*p* > .05) compared with probiotic group (133. mg gallic acid/kg). A concentration‐dependent increase in phenolic content was seen in synbiotic yogurt containing green tea extract, since the phenolic content measured at day 1 was 669.8 and 1,244.9 mg gallic acid/kg at extract concentrations of 0.5% and 1%, respectively. Furthermore, the phenolic content of each treatment group was not statistically affected as a function of storage time (*p* > .05).

### 3.3 Antioxidant activity

**FIGURE 2:**
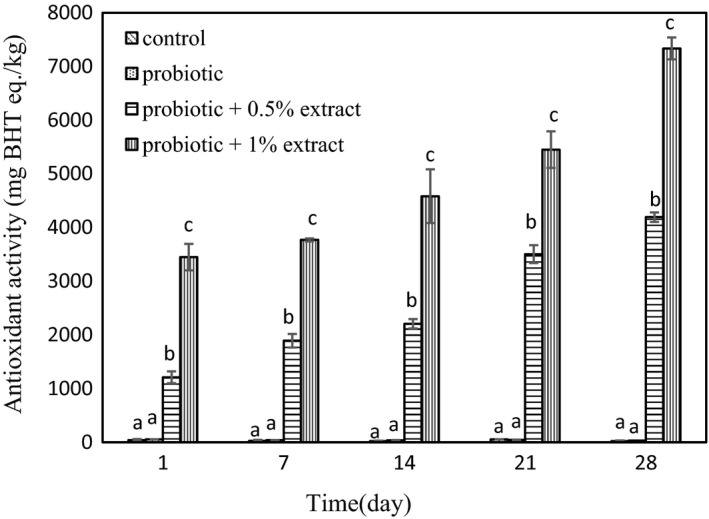
Antioxidant activity of synbiotic yogurt containing aqueous extract of green tea during storage. Different letters at each day show statistical significance between different treatments (*p* < .05)

The results of antioxidant activity of the yogurt samples at different evaluation days are revealed in Figure 2. The antioxidant activity of plain yogurt was estimated 42.3 mg BHT eq./kg at day 1, which remained unchanged during the storage. No statistically significant difference was seen in antioxidant activity of the control probiotic yogurt compared with plain yogurt during 28 days of storage (*p* > .05). Addition of green tea extract to the probiotic yogurt significantly enhanced antioxidant capacity of the yogurt compared with the control probiotic yogurt (*p* < .05). The antioxidant activity of the yogurt was increased as a function of extract concentrations, since synbiotic yogurts containing 0.5% and 1% concentrations of green tea extract exhibited 1,211 and 3,449 mgBHT eq./kg antioxidant activity, respectively. The antioxidant activity of probiotic yogurts containing 0.5% and 1% green tea extract was increased 3.46 and 2.1 times during the storage, respectively.

### 3.4 pH and titratable acidity

**FIGURE 3:**
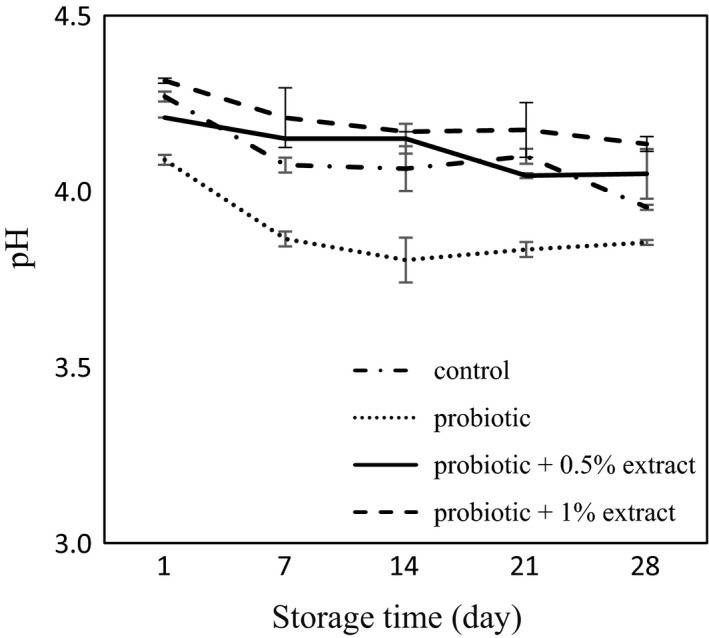
PH value of synbiotic yogurt containing aqueous extract of green tea during storage

**FIGURE 4:**
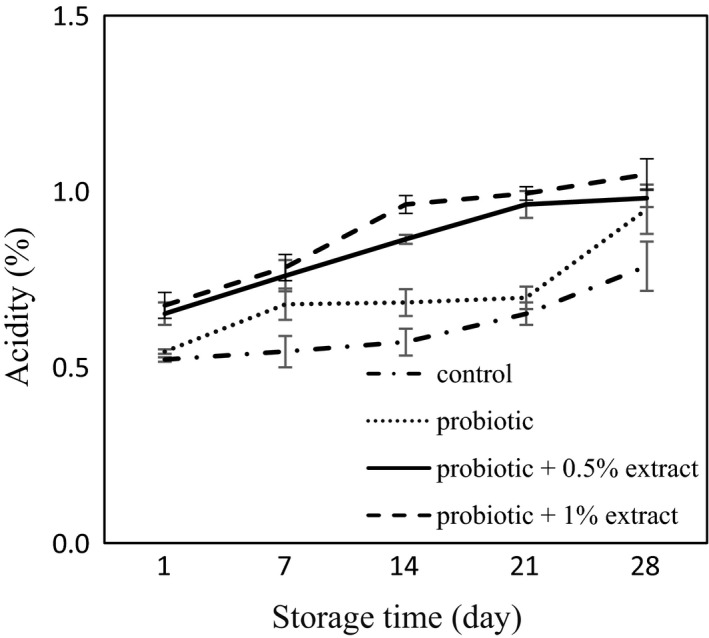
Titratable acidity of synbiotic yogurt containing aqueous extract of green tea during storage

The pH values of yogurt samples are shown in Figure 3. In this study, the pH of the plain yogurt was 4.27 at the first day, which decreased over time and reached 3.95 on day 28. The pH of the probiotic yogurt without extract was reduced from 4.09 at first day to 3.85 on day 28, which showed a significant difference compared with the control group (*p* < .05). No significant changes were seen in yogurt pH with the addition of 0.5% green tea extract compared with the control group, while the pH decreased during the study. In the probiotic yogurt with 0.05% extract, the pH ranged from 4.21 on the first day to 4.05 on the 28th day. By increasing the concentration of the extract to 1%, no significant changes were observed in pH compared to probiotic yogurt with 0.05% extract (*p* > .05). The results of the effect of green tea extract on acidity in probiotic yogurt are presented in Figure 4. In the control group, acidity was calculated to be 0.52% on day 1, which increased to 0.79% on day 28. Although there was no significant difference in the level of acidity in probiotic yogurt compared with plain yogurt on the first day, the acidity increased in probiotic yogurt during the storage so that it was 0.94 on day 28, which showed a significant difference with the control plain yogurt (*p* < .05). Adding 0.5% green tea extract increased acidity compared with control yogurt group. In this group, acidity ranged from 0.65 on the first day to 0.98 on day 28 of the storage. With increasing concentrations of extract to 1%, no significant changes were observed in acidity compared with 0.5% extract contained yogurt (*p* > .05).

### 3.5 Syneresis

**FIGURE 5:**
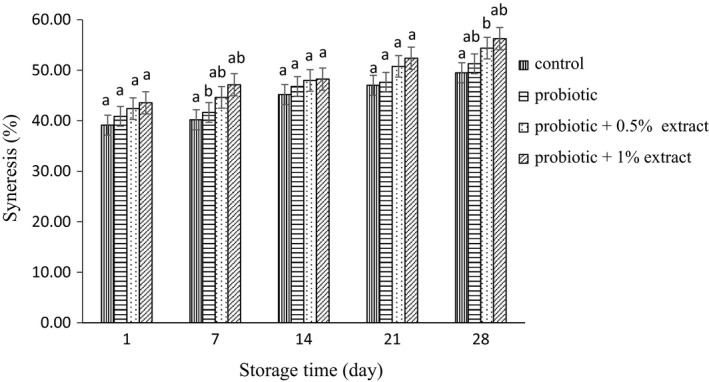
Syneresis of synbiotic yogurt containing aqueous extract of green tea during storage. Different letters at each day show statistical significance between different treatments (*p* < .05)

The results of the effect of aqueous extract of green tea on syneresis in probiotic yogurt with extract (0.05% and 1%) are shown in Figure 5. In the control group, the amount of syneresis was calculated to be 39.13% on the first day, which increased over time and reached 49.5% on day 28. There was no significant difference in the amount of syneresis in probiotic yogurt without extract compared with plain yogurt (*p* > .05). During this study, the amount of syneresis in probiotic yogurt increased to 51.28% on day 28. Adding 0.5% of green tea extract increased syneresis compared with control group. In this group, the rate of syneresis ranged from 42.40% on day 1 to 54.38% on day 28. There were no significant differences in syneresis with increasing concentration of extract to 1% (*p* > .05).

### 3.6 Organoleptic assessment

The results of the effect of the green tea extract on the sensory properties of the probiotic yogurt are presented in Table 3. There was no significant difference in yogurt appearance between the control group and the probiotic yogurt without extract (*p* > .05). By adding 0.5% aqueous extract of green tea to yogurt, the appearance of the yogurt became darker and its appearance acceptance decreased. Increasing the concentration of the extract to 1% increased this color change so that the appearance score in this group decreased to 3.4. No significant difference was found between the taste of the control group and the probiotic yogurt without extract (*p* > .05). While adding 0.5% of the green tea extract to yogurt, the taste of yogurt changed to the sour taste of the extract. With increasing concentration of extract to 1%, the taste of the extract became more intense. No significant difference was found between the texture score in the control group and the probiotic yogurt (*p* > .05), and these groups received the highest score in the textures, which was 5 and 4.9, respectively. Adding 0.5% and 1% of the aqueous extract of green tea reduced the viscosity of yogurt, and yogurt texture became softer. Generally, there was no significant difference between the overall acceptance score of the control group and probiotic yogurt (*p* > .05), since these groups scored 4.8 in overall acceptance. The probiotic yogurt group with 0.5% green tea extract received a score of 3.9 in overall acceptance and yogurt with 1% extract, received the lowest overall acceptance rated 3.2.

**TABLE 3:** Sensory evaluation of synbiotic yogurt containing aqueous extract of green tea during storage

Treatments	Sensory attributes			
	Appearance	Taste	Texture	Overall acceptability
Control	4.9 ± 0.14^a^	5.0 ± 0.12^a^	5.0 ± 0.14^a^	4.8 ± 0.10^a^
Probiotic	4.8 ± 0.28^a^	4.9 ± 0.14^a^	4.9 ± 0.21^a^	4.8 ± 0.28^a^
Probiotic + 0.5% extract	4.1 ± 0.14^ab^	4.3 ± 0.28^ab^	4.0 ± 0.22^ab^	3.9 ± 0.14^ab^
Probiotic + 1% extract	3.4 ± 0.35^b^	3.2 ± 0.28^b^	3.3 ± 0.14^b^	3.2 ± 0.21^b^

Note

Different superscript letters at each column show statistical significance between different treatments (*p* < .05).

## 4 DISCUSSION

In this study, the survival of probiotic *L. acidophilus* and *B*. *bifidum* was investigated in synbiotic yogurt containing aqueous extract of green tea. The results showed that the green tea extract had no adverse effect on the survival of probiotic bacteria. The results of the present study are in agreement with Jaziri et al. (2009), which reported that green tea and black tea did not have any effect on lactic acid bacteria in yogurt during storage. Almajano et al. (2008) also demonstrated the inhibitory effects of green tea, white, black, and red tea infusion on food pathogens, while *L. acidophilus* showed high resistance to all of the studied extracts. In a study by Najgebauer–Lejko (2014), he examined the effects of 5%, 10%, and 15% green tea infusion on probiotic bacteria including *L. acidophilus* and *B. lactis* in probiotic fermented milk and probiotic yogurt. They found that in general the count of Bifidobacteria remained at a higher level for a longer period of time in fermented milk enriched with green tea. However, their results showed that the survival of *L. acidophilus* was negatively dose dependent at concentrations higher than 5%. Also, Noori, Khaji, et al. (2017); Noori, Noudoost, et al. (2017) demonstrated that the use of encapsulation of green tea had a significant role in increasing the survival of probiotic bacteria. It is reported that due to the ability to metabolize phenolic compounds by some lactobacilli species, their survival is related to the amount of total phenol (Lee et al., 2006). Also, according to Tabasco et al. (2011), the sensitivity of Lactobacilli and Bifidobacteria to phenolic compounds depends on the species and bacterial strains, the chemical structure, and the concentration of polyphenol. In their study, the growth of *L. acidophilus* strain was negatively influenced by the addition of grape seed extract enriched with flavan‐3‐ol. In general, the probiotic count of yogurt samples at day 28 investigated in the present study was higher than 10^7^ cfu/g, which was more than the minimum concentration recommended for functional probiotic products (Rybka & Kailasapathy, 1995). In the present study, the addition of green tea extract to yogurt increased the phenolic content. It may be due to the presence of phenolic compounds including epigallocatechin gallate, epigallocatechin, epicatechin gallate, and epicatechin in green tea. Anesini et al. (2008) reviewed the antioxidant activity and phenolic content of different tea in Argentina. The results of their study showed that green tea had the highest phenolic content, and also, there was a direct link between phenolic activity and antioxidants capacity. Furthermore, in the present study, the plain control yogurt revealed some antioxidant activity. It may be due to the presence of bioactive peptides in milk. In addition, proteolysis by lactic acid bacteria results in the release of bioactive peptides with antioxidant activity in fermented milk products (Farvin et al., 2010). Furthermore, the results of this study showed that adding green tea to yogurt increased the antioxidant activity. Tea catechins and some molecular polyphenols can contribute to the high antioxidant potential of green tea, which can explain the high antioxidant activity in tea contained yogurt (Muniandy et al., 2016; Najgebauer–Lejko, 2014). This antioxidant potential of green tea has been proven in various studies. Lee and Sher (1984) showed that green tea leaf extract had good antioxidant properties in soybean oil, which was comparable to BHT. Nieto et al. (1993) studied the role of flavonoids in fish oil stabilization in comparison with several synthetic antioxidants. Their studies showed that catechin flavonoids, quercetin, and moorin in tea leaf had higher antioxidant activity than BHA and BHT. In the present study, a significant correlation was seen between increase in phenolic content and enhancement of antioxidant activity of the yogurt containing different concentrations of green tea extract. It is well documented that polyphenol present in the composition of the plant preparations play an important role in antioxidant activity of them (Muniandy et al., 2016; Vasco et al., 2008). Several studies reported the increase in antioxidant capacity of the yogurt through fortification by pomegranate peel extracts (El‐Said et al., 2014) encapsulated grape seed extract, (Yadav et al., 2018), *Citrus aurantium* L. Flowers (Hashemi et al., 2016), stevia (de Carvalho et al., 2019), Fuzhuan brick‐tea (Liu, 2018), saffron (Gaglio et al., 2019), chickpea water extract (Shori & Baba, 2013), soybean (Shori, 2013), basil and savory (Mosiyani et al., 2017), cinnamon (Shori & Baba, 2011), soy (Shori, 2013a), and chickpea (Shori, 2013b). In this study, the antioxidant activity of yogurt supplemented with an aqueous extract of green tea increased during the storage. It may be due to the degradation of green tea phenolic compounds resulted from continued microbial growth and the production of compounds with higher antioxidant activity and/or the interaction of the compounds derived from milk proteolysis with phenolic compounds of the extract, which increases the antioxidant activity (Joung et al., 2016). Furthermore, the results of the present study showed that acidity increased during storage, which is consistent with earlier researches, which showed increase in acidity of yogurt during the storage (Mosiyani et al., 2017; Najgebauer‐Lejko et al., 2011; Ramirez‐Santiago et al., 2010; Shokery et al., 2017). The probiotic yogurt showed the higher acidity compared with the control plain yogurt. It may be due to the fermentation ability of the probiotic bacteria that resulted in furthers lactic acid production. Furthermore, the probiotic yogurt containing green tea extract exhibited more acidity compared with the control yogurt. This result is in consistence with Amirdivani and Baba (2011) that reported that the addition of plant extracts significantly increased acidity compared with the control group and stated that this may be due to the increased metabolic activity of yogurt bacteria and/or probiotic bacteria in fermented milk with herbal extracts. The results of our study showed that the addition of green tea extract increased syneresis compared with the control group. Although the phenomena that occur during syneresis are not completely understood, but increase syneresis are usually due to the reconstruction of caseins that cause whey to escape (Zare et al., 2011). The finding of the present study was similar to that reported by several researches, which demonstrated increase in syneresis of the yogurt upon incorporation with different plant preparations including olive, garlic, onion, and citrus extract (Michael et al., 2010), *Auricularia auricula* aqueous extract (Faraki et al., 2020), sour cherry pulp (Sengul et al., 2012), and *Pachyrhizus erosus L* fibers (Ramirez‐Santiago et al., 2010). The increase in whey expulsion from the yogurt may be explained by weakening casein network due to interaction with active groups of the extract, thermodynamic incompatibility between polysaccharide of the extract and milk proteins, and unbalanced osmotic potential due to depletion flocculation of the casein micelles in the presence of nonadsorbing polymers such as dietary fiber (Michael et al., 2010). The polyphenol–protein interaction which played an important role in serum separation depends on various factors including proteins and polyphenol nature, temperature, and presence of other bioactive compounds (Vital et al., 2015). However, an improvement of whey separation from the yogurt was reported by addition of *Lactarius volemus* Fr extract (Huang et al., 2020), Jerusalem artichoke powder (Guo et al., 2018), and mango (Narayana & Gupta, 2013) to the yogurt. Also, in a study by Amirdivani and Baba (2013) that used 2% of the Malaysian and Japanese green tea extracts in yogurt, syneresis was increased during the study, but the groups containing green tea extracts showed less syneresis than control group. In the present study, it was observed that the syneresis during storage increased, which is consistent with the results obtained by Ramirez‐Santiago et al. (2010). Whey separation during the storage resulted from protein rearrangement, which weaken the casein network (Everett & Mcleod, 2005). Sensory results of this study showed that probiotic yogurts containing 0.5% and 1% extracts of green tea were less acceptable due to low appearance acceptance, which resulted from discoloration and syneresis, and disturbing the yogurt taste. Meanwhile, in a study by Amirdivani and Baba (2013) that examined the differences between the Malaysian and Japanese green tea extracts in yogurt, panelists preferred yogurt with 2% Malaysian and Japanese green tea extract in terms of appearance, color, and odor in comparison with plain yogurt which in fact reflects the different taste of people in different countries. They reported the amount of acid production during fermentation as one of the factors influencing the taste of yogurt. In their research, simple yogurt texture received a higher score from the panelists, which is consistent with the results of the present study. Shokery et al. (2017) also examined the effects of green tea and Moringa leaf extracts in yogurt and found that yogurt containing green tea extract received less sensory scores than simple yogurt. It is reported that the encapsulation of green tea extract could improve sensory acceptance of synbiotic ice cream (Noori, Khaji, et al., 2017; Noori, Noudoost, et al., 2017). Generally, all the treatments studied in this study were overall acceptable to the panelists.

## 5 CONCLUSION

The present study demonstrated that supplementation of the yogurt effectively increases he total phenolic content and antioxidant activity of the yogurt. Interestingly, the presence of probiotic bacteria induced an intensive increase in antioxidant activity of the yogurt supplemented with green tea extract. Furthermore, the survival of the studied probiotics was not affected by adding the green tea extract. Increased acidity of the green tea extract supplemented yogurt revealed that green tea extract not only had no negative effect on the fermentation process, but also increased acid production ability of the bacteria. Although the green tea extract impaired some sensory properties of the yogurt, the overall acceptability of these treatments remained acceptable yet. In conclusion, according to the results of the present work, simultaneous application of green tea extracts and probiotic bacteria in yogurt is recommended as an effective functional food formulation to improve consumer health.

## CONFLICTS OF INTEREST

The authors declare that there are no conflicts of interest.

## AUTHOR CONTRIBUTIONS

**Fatemeh Rahmani:** Data curation (supporting); Formal analysis (supporting); Methodology (equal); Visualization (equal); Writing‐original draft (equal). **Hassan Gandomi:** Conceptualization (lead); Data curation (lead); Methodology (equal); Software (equal); Supervision (lead); Visualization (lead); Writing‐original draft (lead). **Negin Noori:** Formal analysis (equal); Writing‐review & editing (supporting). **Azita Faraki:** Data curation (supporting); Formal analysis (supporting); Methodology (supporting). **Melika Farzaneh:** Data curation (supporting); Formal analysis (supporting); Methodology (supporting).

## ETHICAL APPROVAL

This study does not involve any human or animal testing.

## Data Availability

The authors confirm that the data supporting the findings of this study are available within the article.
